# Association between HIV status and depressive symptoms among children and adolescents in the Southern Highlands Zone, Tanzania: A case-control study

**DOI:** 10.1371/journal.pone.0193145

**Published:** 2018-02-22

**Authors:** Abraham Lwidiko, Stephen Matthew Kibusi, Azan Nyundo, Bonaventura C. T. Mpondo

**Affiliations:** 1 Department of Mental Health, Ilembula Institute of Health and Allied Sciences, Njombe, Tanzania; 2 School of Nursing and Public Health, College of Health Sciences, the University of Dodoma, Dodoma, Tanzania; 3 School of Medicine and Dentistry, College of Health Sciences, the University of Dodoma, Dodoma, Tanzania; Stellenbosch University, SOUTH AFRICA

## Abstract

**Background:**

Children and adolescents continue to have HIV/AIDS in southern Saharan Africa. Scaling up of HIV services has significantly improved access to ARV and consequently improved on morbidity and mortality related to HIV/AIDS including opportunistic infection. Despite the above efforts, non-communicable conditions including mental disorders such as depression have been observed to contribute to the burden of disabilities about which little is documented. This study, therefore, aimed to determine the magnitude of depressive symptoms and the associated factors among HIV-infected children and adolescents.

**Methods:**

The study was a matched case-control design involving 300 cases of HIV-infected children matched by age and sex against 600 uninfected controls. Systematic sampling technique was used to select the cases while multistage sampling technique was employed to identify villages/ streets purposive and sampling technique was employed to obtain participants from households.

**Results:**

The overall prevalence of depressive symptoms among the cohort of 900 participants was found to be 12.9%, with 27% of HIV-infected and 5.8% of HIV-uninfected children and adolescents screened positive for depressive symptoms. Multiple logistic regression revealed that being HIV-infected (AOR 1.96(1.11–3.45)), residing in a rural setting (AOR 0.61(0.39–0.96)) and history of childhood deprivation (AOR 4.76 (2.79–8.13)) were significantly associated with depressive symptoms.

**Conclusion:**

HIV infected adolescents are more affected by depression compared to non-infected counterparts. Childhood deprivation was significantly associated with presence of depressive symptoms. Integration of mental health evaluation and treatment into the HIV care provided for adolescents can be beneficial. More studies to delineate factors associated with depressed adolescents with HIV may add value to the body of knowledge and overall improvement of care.

## Introduction

HIV/AIDS remains a public health concern in sub-Saharan Africa (SSA). In 2015, 19 million people were estimated to be living with HIV in the region, and 9% of them were children [1]. It has also been reported that out of the 220,000 new pediatric infections globally, 190,000 (86%) occurred in SSA [1]. Although HIV services have been scaled up through increasing uptake of counseling and testing, interventions to prevent mother-to-child transmission of HIV, adherence to AIDS treatment and support and care initiatives for orphaned children such that at least 30% of children living with HIV in SSA are now receiving antiretroviral therapy (ART) [1].

In Tanzania, the government, has strengthened efforts to improve care and treatment services aiming to eliminate MTCT and reach 90% of all pregnant women with treatment, reduce the MTCT rate to less than 5%, and maternal and child mortality by 90% by 2017. Out of 86% of pregnant women living with HIV currently, are receiving effective ART [2,3] and in 2014 a total of 38,848 children living with HIV infection were on ART in 1,209 health facilities throughout the country [4]. Consequently, there has been a significant reduction in morbidity and mortality associated with opportunistic infections [5]. Meanwhile, a rising trend of non-infectious conditions has been observed among children and adolescents with HIV [6].

The World Mental Health survey suggests that there may be a high magnitude of the common mental illnesses such as depression and substance abuse in African countries [7]. Estimates show that HIV and mental illness (depression in particular) will be among the top ten causes of morbidity in developing countries by the year 2030 [8]. Depression has been shown to be the most common mental disorder globally ranking fourth among the leading causes of disease burden and accounting for 3.7% of total disability adjusted life-years and 10.7% of total years lived with disability [9].

Evidence shows that there is significant comorbidity between HIV/AIDS and mental disorders [10,11]. The prevalence of depression among people living with HIV/AIDS (PLWHA) is estimated to range between 12% and 60% [11–13]: however, most of these studies involve adult populations, and very few studies report the magnitude of depression among children and adolescents [14–17]. The few reported studies show a high prevalence of depression among HIV-infected children [15,16] and report several factors associated with depression such as increasing age and female gender (especially after puberty).

Further, evidence suggests that being deprived can increase the risk of mental health problems in children and young people, which in turn can have long-term consequences for their educational and social relationship [18] Children growing up in extreme poverty and deprivation of basic needs are more likely to suffer a wide range of behavioral and emotional problems. One in 6 children in families with low incomes suffers from mental health problems compared to just over one in twenty in a better-off household [19]The relationship between childhood deprivation, HIV status, and childhood depression is not well documented particularly in areas with a high burden of HIV/AIDS.

In light of a limited number of studies that examine the magnitude and factors associated with depressive symptoms in Tanzania, our study, therefore, intended to determine the association between HIV status and depressive symptoms controlling for childhood deprivation among children and adolescents aged between 7 to 17 years in high prevalence and low resource setting.

## Methods

### Study design

A matched case-control study design was employed. The cases were HIV-infected children matched by age and sex against uninfected controls at a ratio of one case to two controls.

### Study setting

The study was conducted in the Mbarali District located in the Southern Highland Zone of Tanzania, Mbeya region. The District consists of twenty (20) administrative wards bordered to the North-east by the Iringa and Njombe regions, to the South by the Mbeya Rural District, and to the West borders the Chunya District. According to the National Population and Household Survey [20], the Mbarali District has a population of 300,517 people; 145,867 males and 154,650 females. More than 72,000 of the total population are children aged above six years. HIV prevalence in the district is estimated to be 12% equivalent to 16,000 adults living with HIV in the district [21], making it one of the areas with the highest burden of HIV/AIDS in Tanzania.

### Study population

Cases were children and adolescents living with HIV/AIDS and attending services at HIV Care and Treatment Centres (CTC) located in the district. HIV status was confirmed by verifying information recorded in the clinic register. Controls were children with known HIV negative status after being verified in Counseling and Testing registers and children with unknown HIV status who were confirmed not being in the HIV testing register. Information of children without a record of HIV infection was further verified at the household by the Researcher, by asking the caregivers and consulting available evidence from available medical cards that each child included in the study had. Children without a clear record of their medical history were not included in the study. Controls were, therefore, children and adolescents identified in the community whose HIV status was either confirmed to be negative or verified to be unknown but assessed to have no history of any chronic illness since birth.

### Sampling and sampling procedure

Using the formula for matched case-control studies [22], A sample of 300 HIV-infected cases and 600 non-HIV-infected case controls were included in the study. The 300 cases were obtained from 15 healthcare facilities offering CTC services to people living with HIV/AIDS in Mbarali District, and the 600 control participants were recruited at the household level.

**Fig 1:**
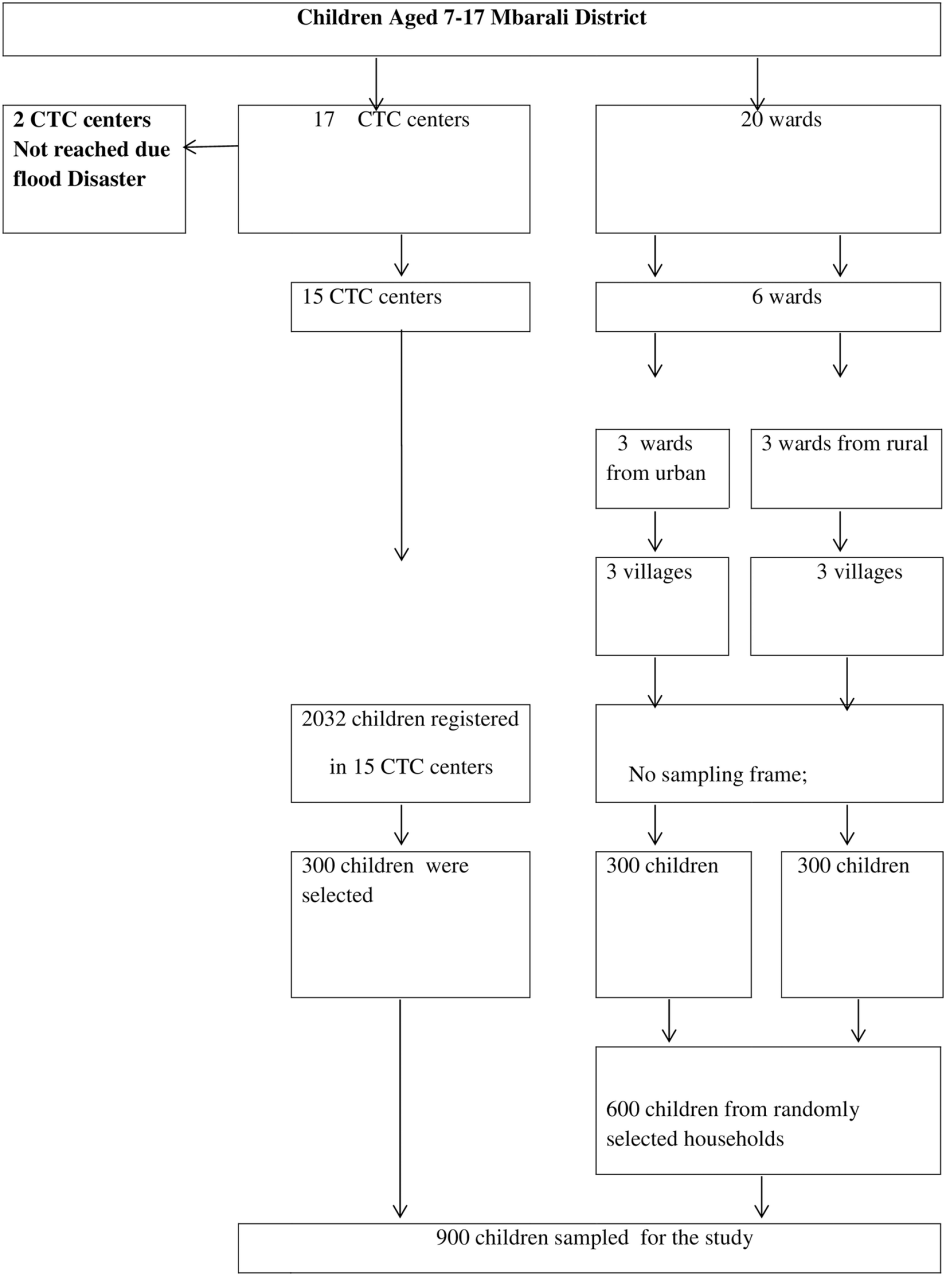
This is the Fig 1 flow chart showing the enrollments of participants for the study at Mbarali District.

Sampling was conducted in two phases; during the first phase, selection of the cases which started with obtaining the sampling frame at the District AIDS Coordinator Office. The second phase involved identification and selection of the Treatment and Care Centers, visiting the centers and we used of the treatment register to systematically select children for the interview as they come to the clinic for their services. At the end a total of 300 children living with HIV/AIDS registered and receiving care at CTC were included in the study. The second phase involved sampling of the controls; a multi-stage sampling was used in selecting Wards from the District, then selection of villages/hamlets and ultimately selecting the streets. At the street level, households were chosen randomly, where a starting point was picked randomly from the street map, the nearby household was visited to identify a child who matched a case by age and sex as well as meeting the inclusion criteria, i.e., HIV negative status or if unknown having no evidence of a chronic illness since birth. If appropriate control was not found in the first house, the next house was visited until the appropriate control was found. To be sure that the control was not HIV infected, the name of the control was verified if it is not in the database of the HIV-infected children from the DAC. If the name was found in the database, the child was not included in the study. Also, an adult caregiver was interviewed to determine whether the child had a history of the chronic disease since birth, responses of the caregivers were verified by checking the medical card of the child. Children without medical cards showing their treatment history were not included in the study. In the end, a total sample of 600 randomly selected children with HIV negative status or unknown status but with no history of any chronic illness after assessment, matching the cases by age and sex were included in the study. The sampling process and enrollment of study participants is summarized in Fig 1.

### Inclusion and exclusion criteria

HIV-infected and uninfected children aged from 7–17 years residing in Mbarali District for whom their parents provided written consent were enrolled in the study. Children lacking registration at CTC (for the cases) and medical cards (controls) were excluded in the study.

### Data collection and data collection tools

Data collection was performed from 16^th^ March 2016 to 18^th^ May 2016. Among the tools used for data collection were a socio-demographic questionnaire, Children Depression Inventory II (CDI II) and WHO clinical staging guideline of HIV/AIDS for children with confirmed HIV infection.

#### Measures and instrumentation

**Children Depression Inventory II (CDI II):** In assessing depressive symptoms among participants, we adopted CDI II scale, which was first translated in Swahili from English and validated to be used in Tanzania in 2010 [23]. It is a psychological measure with a 27 item self- rating scale designed to assess cognitive, affective and behavioral symptoms in children and adolescents. The tool also produces subscale to identify interpersonal problems, ineffectiveness, anhedonia, and negative self-esteem. It is the most widely used self-report measure of depression in children worldwide with normative data available from psychiatric, pediatric and school-based populations. Of the 27-item rating scale, each item is scored on a three-point scale 0-absent, 1-moderate, and 2-severe. The scoring ranges from 0–54 points, with a cutoff point of 12 points as the threshold discriminating children at risk of depression from non-depressed children within homogeneous samples.

### Study measures

#### Depressive symptoms

This was defined as the presence of any two or more simultaneous symptoms of depression present for most or all of the time, at least for two weeks duration associated with evidence of social dysfunction, occurring in individuals who do not meet criteria for minor depression, major depression or dysthymia [24]. Depressive symptoms were assessed using the Children’s Depression Inventory (CDI) which is a comprehensive psychological measure with a 27–item self- rating scale designed to assess cognitive, affective and behavioral symptoms components for youth ages 7–17. This tool has been validated for use in Tanzanian children and adolescents [23].

#### Socio-demographic variables

These were collected using a designed questionnaire. It included child’s characteristics such as the age, sex, area of residence, HIV status and WHO clinical stage of HIV for those with HIV. It also included information about the caregiver such as the level of education, employment status and the relationship of the caregiver to the child.

#### Childhood deprivation

These measures were adopted from the UNICEF General Assembly’s definition of Child poverty. Childhood deprivation is defined as lack of access to nutrition, water and sanitation facilities, access to basic health care services, shelter, education, participation, and protection leaving children unable to enjoy the right to reach their full potentials as members of the society (UN General Assembly, 2007). In this study, the Global Multidimensional Poverty Index (MPI) was used in measuring childhood deprivation. MPI is a measure of poverty designed to capture the multiple deprivations that each household, faces at a given point in time with respect to education, health and other aspects of living standards (Andersen et al., 2015). The MPI index was developed by Alkire and Foster 2007, 2011(Andersen et al., 2015) using data from USAID demographic and Health Surveys (DHS), and the UNICEF-Multiple indicator cluster surveys (MICS). These unmet needs result in children who are unable to reach their potential and participate as full members of the society [25].

### Data management and analysis

Data were coded and entered into a computer database and cleaned before data analysis. Data were analyzed by using SPSS 20 version. Chi-square analysis was done to determine group differences in the outcome variable. The level of significance was set at P<0.05 (2-tailed) for all the analyses. Both bivariate and multiple logistic regression were used to generate crude (OR) and adjusted odds ratios (AOR). Odds ratios were estimated to assess the strength of the associations and used the 95% confidence intervals (CIs) for significance testing. All the covariates were entered simultaneously into the multiple regression models.

### Reliability of the CDI II scale

Following validation, CDI II scale scored an alpha estimate of 0.669 with internal consistency reliability estimate 0.109 to 0.575 which was within normal range acceptability when employing community as per original constructs [23]. The tool has been used in Tanzania in the regions of Mbeya, and Dar es Salaam where two different studies have been conducted. The CDI II scale is also routinely used at Muhimbili National Hospital Psychiatry Department in Dar es Salaam, Tanzania.

### Ethical consideration

Ethical clearance was obtained from the University of Dodoma Research Publication Committee. Permission to conduct a study was granted by the District Executive Director, Mbarali district. Participants were thoroughly informed about the study; since the participants were below the age of 18 and only those participants whose parents or guardians provided written consent were included in the study. Confidentiality was guaranteed through participant’s state of anonymity as participants were identified through numbers but not names. Participants’ rights such as freedom to withdraw from the study, not answering some questions and other rights were addressed and observed.

## Results

### Baseline characteristics of study participants

A total of 900 children in Mbarali district were enrolled in the study. Of these, 300 (33.3%) were HIV-infected (the cases), and 600 (66.7%) were uninfected (the controls). The mean age of the respondents was 12.84 years (S.D 2.2). Of the participants recruited, females constituted 174 (58%) of cases and 331 (55.2) of controls within the study population. The majority 198 (66%) of HIV-infected children were living in an urban setting. However, among the sample from the general population, the participants were evenly selected from rural and urban settings and each constituted 300 (50%) respectively. Thirty-six (12%) and 11 (1.8%) of the HIV infected and uninfected children respectively reported to have lost both parents, 99 (33.0%) of the HIV infected children reported to be living with a single parent while only 63 (10.5%) of the children in control group reported to be living with a single parent. See Table 1.

**Table 1:** Socio-demographic characteristics of HIV infected and uninfected children Mbarali District.

Variable		Case (n = 300)	Control (n = 600)	X^2^(df)	P- Value
**Sex of Respondent**	Male	126 (42)	269 (44.8)	0.65(1)	0.434
	Female	174 (58)	331 (55.2)		
**Level of Education of a Care Giver**	None	50 (16)	17 (2.8)	86.8(3)	0.00
	Primary school	220 (73.3)	403 (67.2)		
	Secondary school	22 (7.3)	133 (22.2)		
	Higher education	8 (2.7)	47 (7.8)		
**Place of Residence**	Urban	102 (34.0)	300 (50)	20.72(1)	0.00
	Rural	198 (66.0)	300(50)		
**Employment Status of a Care Giver**	Employed	36 (12.0)	210 (35.0)	106.24(2)	0.00
	Self-employed	171 (57.0)	342 (57)		
	Unemployed	93 (31.0)	48 (8.00		
**Type of a Care Giver**	Parents	63 (21.1)	401 (66.8)	173 0.53(3)	0.00
	Single parent	99 (33.0)	63 (10.5)		
	Guardian	79 (26.4)	79 (13.2)		
	Orphanage Centre	59 (19.7)	57 (9.5)		
**WHO clinical staging of HIV confirmed children**	Clinical stage 1	219 (73.0)	NA		
	Clinical stage 2	33 (11.0)	NA		
	Clinical stage 3	21 (7.0)	NA		
	Clinical stage 4	27 (3.0)	NA		

### Factors related to the prevalence of depressive symptoms among children in Southern Highland Zone, Tanzania

The prevalence of depressive symptoms was found to be present in 27% of HIV-infected and 5.8% of HIV-uninfected children and adolescents. The overall prevalence of depressive symptoms in this cohort of 900 participants was found to be 12.9%. The study found that positive HIV-status (X^2^ = 79.8, p-value <0.001), rural residency (X^2^ = 12.7, p-value <0.001), unemployment status of caregivers (X^2^ = 25.7, p-value <0.001), lack of formal education of the caregiver (X^2^ = 25.1, p-value <0.001), living with single parent (X^2^ = 30.5, p-value <0.001) and deprivation status (X^2^ = 119.0, p-value <0.001) all had a significant relationship with depressive symptoms, (Table 2). Results from multiple logistic regression showed that; being HIV-infected (AOR 1.96(1.11–3.45)), residing in a rural setting (AOR 0.61(0.39–0.96)) and being deprived (AOR 4.76 (2.79–8.13)) were significantly associated with having depressive symptoms (Table 3).

**Table 2:** Univariate analysis for factors related to depressive symptoms among children aged 7–17 years in Southern Highlands Zone, Tanzania (X^2^ n = 900).

Variable		Depressive symptoms		X^2^	P-value
		Screened positive	Screened negative		
**HIV status**	Positive	81 (27)	219(73)	79.81	<0.001
	Negative	35(5.8)	56.5(94.2)		
**Age-group**	7–12	51(15.3)	283(84.7)	2.68	0.102
	13–18	65(11.5)	501(88.5)		
**Sex of respondent**	Male	47(11.9)	348(88.1)	0.615	0.433
	Female	69(13.7)	436(86.3)		
**Residence of the respondent**	Urban	34(8.5)	368(91.5)	12.70	<0.001
	Rural	82(16.5)	416(83.5)		
**Employment Status of the Parent/Guardian/Caregiver**	Employed	17(6.9)	229(93.1)	25.74	<0.001
	Self-employed	64(12.5)	449(87.5)		
	Unemployed	35(24.8)	106(75.2)		
**Education status of the caregiver**	No formal	18(26.9)	49(73.1)	25.07	<0.001
	Primary	87(14.0)	536(86.0)		
	Secondary	11(7.1)	144(92.9)		
	College/University	0	55(100)		
**Type of caregiver**	Parents	35(7.2)	450(42.8)	30.50	<0.001
	Single	26(19.8)	105(81.2)		
	Grandparent	33(20.4)	129(79.6)		
	Orphanage	22(18.0)	100(82.0)		
**Deprivation status**	Deprivation	75(34.6)	142(65.4)	119.00	<0.001
	No deprivation	41(6.0	642(94.0)		

**Table 3:** Crude and adjusted odds ratio for the association between HIV status and depressive symptoms among children aged 7–17 years in Southern Highland Zone, Tanzania (N = 900).

Variable		Unadjusted OR (95%CI)	Unadjusted p-value	Adjusted OR (95%CI)	Adjusted P-value
**HIV status**	HIV-infected	3.97(9.90,9.14)	0.00**	1.96 (1.11–3.45)	0.02*
	HIV-uninfected	1		1	
**Employment status of caregivers**	Employed	0.52(0.30,0.91)	0.02*	1.76 (0.87–3.53)	0.11
	Self employed	0.23(0.12,0.42)	0.00**	1.52 (0.91,2.54)	0.11
	Unemployed	1		1	
**Deprivation status**	Deprived	8.27(5.42,12.61)	0.00**	4.76 (2.79–8.13)	0.00**
	Not deprived	1		1	
**Residency**	Urban	0.45(0.31,0.72)	0.00**	0.61 (0.39–0.96)	0.03*
	Rural		1	1	

** = 0.01,

* = 0.05

## Discussion

This study aimed to assess the magnitude and factors associated with depressive symptoms among HIV-infected children compared to age and sex-matched non-HIV-infected controls in Mbarali District. We found that the prevalence of depressive symptoms in HIV-infected children was five times that of age-matched HIV-negative children (27% vs. 5.8%). Although evidence suggests both HIV-infected and HIV-affected children and adolescents face serious mental health challenges [26], few studies have documented the magnitude and the risks for psychiatric disorders such as depression among HIV infected children and adolescents in SSA. Numerous studies have demonstrated a prevalence of depressive symptoms among HIV-infected children [15,16,27]. A study conducted in Uganda showed a 21% prevalence of depressive symptoms among school going HIV-infected adolescents [27] which is in line with other studies conducted in both the global north and global south [28–30].

As reported by studies elsewhere, the reported prevalence of depression in our study is relatively lower than the prevalence of depression for adult’s counterparts with HIV [31], although aging has been shown to be a strong predictor for depression in HIV population [31]; however, few reports suggest that the risk for depression in adolescents with HIV is four times higher that of their peers in the general population while for the case of adults with HIV while other the risk is reported to be twice that of general adult population [32]. As for the risk factors, inherited risks, developmental factors, sex hormones, and psychosocial adversity interact to increase risk through hormonal factors and associated perturbed neural pathways are major factors with depression in adolescents but not in adults [33].

Our study supports other previous studies that depressive symptoms are more common in HIV-infected than HIV-uninfected individuals [34]. Although a study conducted in Rwanda showed similar odds for depressive symptoms [15], this could partly be explained by the fact that the comparison group was of HIV affected children whom by definition have their parents infected with HIV and thus they may also be at risk for depressive symptoms.

Higher prevalence of depressive symptoms among HIV infected adolescents compared to peer adolescents may partly be due to the direct effect of HIV on the developing brain, possibly the long-term effect of antiretroviral therapies and various biological and social stressors [35]. Furthermore, biological, societal and viral factors may augment each other and potentiate the disease complications of the virus to the developing brain [36].

Our study reveals that childhood deprivation, unemployed caregiver and residing in rural settings to significantly predict depressive symptoms, this observation highlights the link that low socio-economic and socio-cultural factors play a role in the occurrence of depressive symptoms [28,37,38]. There is overwhelming evidence that low socioeconomic status, female gender, low education of the parent, parenting style, and poor academic performance are highly linked to the occurrence of depressive symptoms among adolescents as they may be more exposed to difficult living conditions [28,37,39]. The role of social and emotional childhood deprivation on the pathogenesis of future mental health has been well established; and this study supports the previous observations that childhood deprivation has a significant psychological impact in adolescence and adulthood [40–42].

Although the difference in our study was not significant, similar to previous studies [28,37,38,43], there were relatively more females who suffered from depressive symptoms compared to males. Teenage girls are considered more at risk for mood disorders including depression; the risk is suggested to be the result of distinct biological, social and psychological dynamics [44–46], The lack of association could be explained by the hidden confounders such as age, pubertal status, pubertal timing, and perceived timing about which the analysis of their interaction towards sex differences in adolescent depression was beyond the scope of this study [47–49].

Our study had several limitations. The study enrolled children and adolescents from one district in southern Tanzania. Thus the results may not necessarily be generalizable. Other factors such as school performance, psychosocial determinants, CD4+ count, viral load; comorbid psychiatric conditions which may also associate with depressive symptoms were not taken into account. The HIV status of some children who had never tested was not verified. Our study, however, is one of the few studies in Tanzania to assess the relationship between HIV status and depressive symptoms in children and adolescents using the CDI-II scale that has already been validated for use in Tanzanian children.

## Conclusion

Depressive symptoms are more prevalent among HIV infected adolescents compared to non-infected counterparts. This study highlights the need to integrate mental health evaluation and treatment into the care provided for adolescents with HIV. At a minimum, screening and treatment for depressive symptoms and depression should be considered. If depression is not well managed, it may negatively impact the child’s short and long-term prognosis. Furthermore, additional studies to further delineate factors associated with depressed adolescents with HIV will be highly beneficial.

## Supporting information

S1 Data Set This is the S1 Data Set. (XLS)
